# Influenza B-induced refractory cardiogenic shock: a case report

**DOI:** 10.1186/1471-2334-13-452

**Published:** 2013-09-30

**Authors:** Mahnaz Taremi, Anthony Amoroso, Heather L Nace, Bruce L Gilliam

**Affiliations:** 1 Institute of Human Virology and Division of Infectious Diseases, University of Maryland School of Medicine, Baltimore, MD, USA

**Keywords:** Influenza B, Myocarditis, Cardiogenic shock, Adult, Extracorporeal membrane oxygenation

## Abstract

**Background:**

An association between influenza A viruses and myocarditis was noted during the 1918 influenza pandemic. Since then, the link between the influenza B virus and fulminant myocarditis or cardiogenic shock has been rarely reported.

**Case presentation:**

In February 2013, a 50 year-old-woman without known heart disease presented in profound cardiogenic shock with a left ventricular ejection fraction of 10%. Her presentation was preceded by six days of fever, chills, myalgia and fatigue. She had a junctional tachycardia, a troponin I of 12.6 ng/ml and her coronary angiography demonstrated normal coronary arteries. Percutaneous extracorporeal membrane oxygenation was required. An endotracheal aspirate at admission was positive for influenza B. All other respiratory, blood and urine cultures were negative. On day 7, a repeat echocardiography demonstrated significant recovery of left ventricular function with an ejection fraction of 50%. She was later discharged home in good condition.

**Conclusions:**

Influenza B infection can be complicated by fulminant cardiomyopathy leading to cardiogenic shock in adults without preexisting cardiac disease.

## Background

Myocardial involvement caused by the influenza virus ranges from mild asymptomatic to fulminant myocarditis or cardiomyopathy resulting in cardiogenic shock [1-3].

Influenza induced cardiogenic shock is extremely rare and most reported cases are due to fulminant myocarditis associated with influenza A virus [4-6]. Influenza B virus infection is generally considered to be mild with less associated cardiovascular involvement in adults [7].

Here we describe a woman with influenza B who developed cardiogenic shock with no known pre-existing heart disease. She was successfully managed with aggressive pharmacological and percutaneous extracorporeal membrane oxygenation (ECMO) support.

## Case presentation

The patient, a 52-year-old healthy woman without a significant past medical history, presented to a community care center complaining of 5 days of productive cough, diffuse myalgia, and subjective fever with chills. She was prescribed a course of azithromycin for presumed bronchitis. The following day, she presented to her local emergency department with acute onset of severe shortness of breath and associated chest pain. She was found to be hypotensive (90/60 mmHg), tachycardic (115 beats/min), and was admitted to the intensive care unit (ICU) with a presumptive diagnosis of septic shock. Norepinephrine and empiric IV vancomycin and piperacillin/tazobactam were started. Initial labs revealed: white blood cell count (WBC) 12.7/μL with 77.9% neutrophils, hemoglobin 12.7 g/dl, hematocrit 39.8%, platelets 141, lactate 8.2 mmol/L, sodium (Na) 149 mmol/L, potassium (K) 4.3 mmol/L, bicarbonate 16 mmol/L, chloride (Cl) 115, blood urea nitrogen (BUN) 28, creatinine (Cr) 1.3 mg/dl, blood glucose 214 mg/dl, aspartate aminotransferase (AST) 128 IU/L, alanine aminotransferase (ALT) 200, alkaline phosphatase (AP) 104 IU/L, serum troponin I 12.6 ng/ml, and creatine phosphokinase (CPK) 339 U/L. Her electrocardiogram revealed an accelerated junctional rhythm with a rate of 115 beats/min, poor R wave progression and low voltage. An emergent transthoracic echocardiogram (TTE) showed severely impaired left ventricular (LV) function with an estimated ejection fraction (EF) of 10%. Within 24 hours of presentation her condition deteriorated and she required intubation and mechanical ventilation. The patient then was transferred to our center for further management.

Upon arrival the patient was intubated, sedated, and afebrile. Her blood pressure was 70/40 mmHg and she had a heart rate of 110 beats/min. Examination revealed bilateral crepitations in the lungs, normal heart sounds and a benign abdomen. Her skin was mottled; and her extremities were cold and clammy. There was no pedal edema. Chest radiograph revealed diffuse bilateral patchy infiltrates and pulmonary vascular congestion. A repeat TTE demonstrated a left ventricular ejection fraction of 10%, normal LV and RV dimensions, moderate mitral regurgitation, left ventricle wall thickness of 12 mm, and global hypokinesis of LV with minor regional variation. There was no evidence of a pericardial effusion. Notable labs were a WBC 14.8/μL with 82% neutrophils, hematocrit 36.5%, platelets 113, CPK 365 U/L, troponin 7.6 ng/ml and Cr 0.76 mg/dl. The patient remained hypotensive despite maximum doses of norepinephrine, dobutamine and vasopressin. She was continued on empiric antibiotics and empiric oseltamivir (75 mg twice daily) was added. Immediate left and right heart catheterization demonstrated normal coronary anatomy, with a cardiac index of 1.16 L/min/m2. Emergent venoarterial ECMO (flow = 4 L/min) was placed via left common femoral vein and artery.

On day 3 of hospitalization, pulmonary congestion significantly improved but another repeat TTE showed persistent severe LV systolic dysfunction with an EF of 15%. Endotracheal aspiration at admission returned positive for influenza B by polymerase chain reaction (PCR). Right ventricular endomyocardial biopsy was performed on hospital day 3 (total 4 specimens). Histopathology was negative for inflammation. Immunohistochemical stain for parvovirus B19, HHV6, HSV and PCR for enteroviruses, EBV and CMV were all negative. Bronchoscopy revealed mild mucosal erythema with clear secretions. The bronchoalveolar lavage (BAL) fluid was negative for enteroviruses, adenoviruse, CMV, EBV and HSV by PCR. Blood, BAL and urine cultures were negative for bacterial growth and empiric antimicrobials were stopped on day 5 and oseltamivir was continued for a total of 7 days.

The patient’s clinical status gradually improved, inotropic support was stopped and a repeat TTE on day 7 showed significant recovery of left ventricular function with an estimated EF of 50%, and ECMO support was able to be withdrawn.

The patient was extubated on hospital day 8; low doses of a β -blocker and an angiotensin converting enzyme inhibitor were started. The patient’s course was complicated by a pseudoaneurysm of the left common femoral artery secondary to the ECMO therapy, which was repaired successfully. She was discharged on hospital day 15. Transthoracic echocardiography one month after discharge demonstrated normal LV systolic function with an EF of 60%, normal LV wall thickness, trace mitral regurgitation and no ventricular dilation.

### Discussion

During the 1918–1919 influenza pandemic an association between myocarditis and influenza viruses was noted during an autopsy study [8]. While there have been reports of fulminant myocarditis or cardiomyopathy associated with influenza B in children, it has very rarely been reported in adults [7,9-11]. In 1958, a group of four patients with pericarditis, subacute myocarditis, and fatal chronic myocarditis associated with influenza B were documented [12]. Ray CG et al. described a 34-year-old healthy woman who presented with acute onset of dilated cardiomyopathy and cardiogenic shock following a few days of flu like syndrome. She had a very complicated course and died 6 weeks after the onset of symptoms. Influenza B virus was isolated from the nasopharynx and the diagnosis of acute myocarditis was confirmed by cardiac autopsy [13].

McCarthy et al. described fulminant myocarditis as a distinct clinical presentation of abrupt onset of severe heart failure and cardiogenic shock preceded by a viral syndrome similar to our patient [14]. The typical echocardiographic findings in fulminant myocarditis are described as near normal ventricular dimensions, severely depressed LV systolic function, and increased left ventricle wall thickness [15]. Histological diagnosis of fulminant myocarditis can be difficult especially in the early phase of disease. The limited sensitivity of cardiac biopsy for detection of myocarditis is well recognized and negative cardiac biopsies results cannot exclude the diagnosis of myocarditis [16]. Myocarditis usually presents as a focal or patchy infiltration predominantly in the lateral free wall of the left ventricle and often not involving the right ventricle, therefore, biopsies taken from the right ventricle could be falsely negative [17].

This patient’s clinical presentation of refractory cardiogenic shock 6 days after the onset of influenza and severely impaired left ventricular systolic function, and subsequent normalization of ventricular function after recovery of her viral illness; taken together with her typical echocardiogram findings in the setting of normal coronaries supports the clinical diagnosis of viral fulminant myocarditis; regardless of non diagnostic right ventricle biopsies. During influenza infection, severe myocardial dysfunction can be caused not only by direct injury to cardiac myocytes but also by overexpression of cytokines and severe inflammatory response to viral infection resulting cytokine and humoral mediated forms of myocarditis with no cellular infiltrate.

The reason for the unexpected severity of influenza B infection in our patient was not clear. Clinical data about risk factors and complications of influenza B are limited. Her only past medical history was remote myasthenia gravis and thymectomy over 20 years prior. The predominant circulating B strain was B/Wisconsin/1/2010- LIKE when the patient presented to the hospital, which was matched with the B component of the 2012–2013 vaccine [18]. We do not know if the patient had received an influenza vaccine this season. The possible lack of immunologic memory for this infection might have contributed to the severity of influenza B virus infection in our patient. Increasing awareness among patients and physicians about the importance of vaccination as the most important strategy for minimizing the severe complications of influenza infection could help to prevent similar cases in the future.

The treatment of influenza associated fulminant myocarditis still remains supportive. Studies have shown the early use of mechanical circulatory support for patients with fulminant myocarditis and cardiogenic shock who fail aggressive pharmacologic treatment would increase survival rate [19,20]. Our patient received oseltamivir, standard heart failure treatment but more important early initiation of ECMO support before irreversible organ failure developed.

## Conclusions

In conclusion, we report this case to draw attention that influenza B, which is usually considered less pathogenic, can unexpectedly be complicated by fulminant myocarditis or cardiomyopathy leading to cardiogenic shock in adults. Therefore, a multidisciplinary approach including early initiation of antiviral treatment and aggressive cardiac support is essential for a favorable outcome.

## Consent

Written informed consent was obtained from the patient for publication of this case report. A copy of the written consent is available for review by the Editor-in-Chief of this journal.

## Competing interests

The authors declare that they have no competing interests.

## Authors’ contributions

MT performed a literature review and wrote the manuscript. AA revised critically the manuscript. MT, HLN and AA contributed in her care. HLN and BLG reviewed the manuscript. All authors read and approved the final version of the manuscript.

## Pre-publication history

The pre-publication history for this paper can be accessed here:

http://www.biomedcentral.com/1471-2334/13/452/prepub
